# Comprehensive Identification of Tumor-associated Antigens via Isolation of Human Monoclonal Antibodies that may be Therapeutic

**DOI:** 10.4110/in.2009.9.1.4

**Published:** 2009-02-28

**Authors:** Yoshikazu Kurosawa

**Affiliations:** Institute for Comprehensive Medical Science, Fujita Health University, Toyoake, Aichi 470-1192, Japan.

**Keywords:** phage-display Abs, therapeutic Abs, tumor-associated antigens

## Abstract

Although the success of trastuzumab and rituximab for treatment of breast cancer and non-Hodgkins lymphoma, respectively, suggests that monoclonal antibodies (mAbs) will become important therapeutic agents against a wider range of cancers, useful therapeutic Abs are not yet available for the majority of the human cancers because of our lack of knowledge of which antigens (Ags) are likely to become useful targets. We established a procedure for comprehensive identification of such Ags through the extensive isolation of human mAbs that may be therapeutic. Using the phage-display Ab library we isolated a large number of human mAbs that bind to the surface of tumor cells. They were individually screened by immunostaining, and clones that preferentially and strongly stained the malignant cells were chosen. The Ags recognized by those clones were isolated by immunoprecipitation and identified by mass spectrometry (MS). We isolated 2,114 mAbs with unique sequences and identified 25 distinct Ags highly expressed on several carcinomas. Of those 2,114 mAbs 434 bound to specifically to one of the 25 Ags. I am going to discuss how we could select proper target Ags for therapeutic Abs and candidate clones as therapeutic agents.

## INTRODUCTION

In order to develop therapeutic mAbs against cancers, many groups have been trying to identify tumor-associated Ags (TAAs) using micro array technology (1,2). Afterwards they started to generate mAbs against them. Our approach was designed in the opposite way to this strategy and is quite straightforward. At the first step we isolated a large number of human mAbs that bound to membrane proteins present on cancer cells. At the second step we selected clones that significantly and selectively bound only to tumor cells. At the third step we identified Ags that were recognized by such tumor- specific Abs. Finally we searched for clones that showed strong anti tumor activities *in vitro* and *in vivo*. If this strategy can work well, many researchers in this field will adopt it and do it. However, when we performed experiments in a conventional way, we experienced a couple of problems. In the first process the number of clones with different sequences in the pool of mAbs picked up tended to be limited. Moreover, the affinity of isolated clones to the Ags was not so high as expected. In the third process it has not been easy to comprehensively identify the Ags recognized by respective mAbs because they are membrane proteins. Actually a couple of groups adopted a similar strategy to ours, but they identified only few TAAs (3,4).

## IDENTIFICATION OF TAAs AND SIMULTANEOUS ISOLATION OF HUMAN mAbs AGAINST THEM

One of the main issues to be solved was development of a screening method for comprehensive isolation of mAbs specific against membrane proteins. As a source of Abs we used a phage Ab library constructed from human B lymphocytes (5). In the case of conventional method (6,7), cells and phage particles are mixed. After Ags and Abs form complexes on the cell surface, the cells are collected by centrifugation. Since the complex formation is an equilibrium reaction, we could expect that the Abs with strong affinity to Ags should stay with cells longer than the Abs with weak affinity. However, majority of the clones isolated by this method showed low affinity and appeared to be biased to specific clones. When the researchers obtained these results, they usually thought that since the Abs contained in the Ab library are naive to the Ags, they could not expect the Ab with high affinity to the Ags. We also thought so in the beginning, but gradually started to understand well the characteristics of the phage Ab system. Finally we succeeded in developing the ICOS (isolation of Ag/Ab complexes through organic solvent) method (8). In the case of the ICOS method while the first step for mixing of the cells and phage Abs in an aqueous solution is the same as that in the conventional method, the mixture is put on the organic layer and centrifuged. Therefore the phages complexed with cells via Ag/Ab interaction were collected through organic solution. Majority of the clones isolated by the ICOS method showed good quality in terms of diversity and affinity. We performed 51 time screenings against 33 different cancer cell lines derived from hepatocarcinoma, renal carcinoma, pancreatic carcinoma, lung carcinoma, colonic cancer, gastric cancer and ovarian cancer. We picked up around 200 clones on average in each screening, therefore, 9,395 clones in total. Among them 80% of clones expressed the Ab on the phage particle. Sequence analysis indicated the same clones had been redundantly isolated. Therefore, finally 2,114 distinct clones were isolated in these screenings (9).

At the second step we extensively carried out immunohistochemical analyses using fresh cancer tissues for all of the 2,114 mAbs isolated as above. The results indicated that approximately one third of the Abs isolated by the ICOS method bound to TAAs. Although these observations were striking, that is, the frequency of tumor-specific staining appeared to be very high, much higher than we had expected, we initially doubted whether apparently TAAs are really tumor-specific or some artifacts or misjudgment. Eventually, these results turned out to reflect the true situations.

Since we had mAbs and the cells which expressed the target Ags recognized by respective clones, we imagined, in the beginning, that it may be easy for us to identify the Ags one-by-one through purification either by imunoprecipitation or by affinity chromatography followed by MS. But soon it turned out to be not easy. The difficulty was mainly caused during the process of solubilization by detergent. Furthermore, we had several hundred mAbs. If we had to identify Ags one-by-one, it looked an endless work. In our system a single-chain Fv (scFv)-C_L_ form fused with a truncated cp3 was adopted as the phage-display Ab form. In this molecular form, it will take a long time for us to purify many clones such as several hundreds. But since in our gene form an scFv-C_L_-cp3 can be easily converted to an scFv-C_L_ fused with protein A domains simply by digestion with a restriction enzyme followed by ligation (10). Therefore, we easily purified many clones by using IgG-conjugate column, and then they were used for further analysis. In order to comprehensively identify the Ags recognized by many clones, we newly developed two methods, GFC [grouping of clones by flow cytometry (FCM)] method and SITE [simultaneous identification of clones through three dimensional ELISA (enzyme-linked immunosorbent assay)] method.

When we looked at the patterns analyzed by FCM using various mAbs against some specific cells and compared them, we noticed that some of the patterns recognized by different clones were almost identical to each other. It is likely that they recognized the same molecules or the same complexes. We routinely performed FCM analysis of respective mAbs against six different cells. Based on the similarity of the patterns, the clones were classified into groups and presence of 30 to 40 groups were revealed. We termed this procedure the GFC method. The clones classified into the same group were simultaneously treated for immunoprecipitation followed by MS. Since Ab quite often showed a cross-reactivity, moreover since amounts of membrane protein were generally much smaller than those of cytoplasmic proteins, it is not easy to purify by only one immunoprecipitation. Contaminated cytoplasmic proteins disturbed the MS analyses. Therefore, we biotinylated membrane proteins before immunoprecipitation. By these devices we have succeeded in identification of 25 kinds of TAAs as summarized in Table I (9). They were recognized by 91 mAbs. But we have several hundred more clones that gave cancer-specific staining patterns.

We had 2,114 mAbs with different sequences. While 91 of them were known to bind to one of the above 25 Ags, it was quite likely that there remained many clones that also bound to one of them. Therefore, we prepared the polypeptides that corresponded to the extracellular portion of these TAAs. If we performed ELISA against the 2,114 clones by using these polypeptides as Ags, we could know which clones bind to one of them. But ELISA of 2,114 samples required too much amount of Ags. Therefore we developed the SITE method. All the clones were stored in 96-well plates. Then clones were mixed in three different ways according to plate number, column number and rank number. We performed ELSA of much smaller number of mixed samples than 2,114 each of which was composed of several ten clones. Based on the address numbers of mixtures, plate, column and rank numbers, that gave positive signals, we could know which clones were positive ones. Development of the SITE method enabled us to know that further 343 clones bind to one of the 25 TAAs as indicated in Table I (9).

## REQUIREMENTS FOR THERAPEUTIC TARGET Ags AND Abs

People suspected that since the Abs in the library should be naive to the TAAs, their affinity to the Ags may not be high enough for the therapeutic purpose. Therefore, after identification of new target Ags, they have to start isolating mAbs against them. They also argued that since we started mRNA of B cells present in human bodies as a source of immunoglobulin genes for the construction of Ab library, Abs in the library cannot bind well to the human-originated proteins. According to our experiences, however, both arguments turned out to be wrong. We succeeded in isolation of many kinds of mAbs with high affinity against almost any human-originated TAAs from human B cell-derived Ab library. Comparison of the binding activity of anti EGFR mAb between commercially ERBITUX and our clones indicated that both Abs had almost identical Ag binding activity to EGFR present on the living cell surface, such as 0.1 to 1 nM *Kd* value. We also examined the accumulation of indium-111 labeled anti EGFR Ab to the tumors in nude mice. Our mAb accumulated well to the cancer cells and the degree of accumulation was judged to be a similar level to Erbitux. The Ab-dependent cell-mediated cytotoxicity (ADCC) activity shown by anti EpCAM Ab was shown even at lower than picomolar level. These examples were not exceptional among the several hundred clones that we have isolated. Therefore, the questions that remain to solve for development of therapeutic Abs are how to select TAAs as targets and how to select mAbs for performing clinical examinations.

For development of therapeutic Abs against cancers, we classified the Ags to three categories as summarized in Table II. Against the Ags such as EGFR, HER2 and EpCAM, therapeutic Abs have already been developed. In these cases, it has been already proved that mAbs are effective against cancers. There are only a few Ags among the 25 Ags that were classified into this category. One third of the Ags identified to date were classified into the second category. Although cancer-specific expression was very clear, they have not been considered to be proper targets for therapeutic Abs. In the case of Ags classified into this category their function in tumorigenesis has not been well charcterized. For example, CD73 is an enzyme that mediates as nucleotidase from AMP to adenosine. We obtained the data that anti CD73 Ab completely inhibited cell growth. It is very difficult for us to imagine why the change from AMP to adenosine in the environment of cancer cells is required for growth of cancer cells. Two thirds of the Ags were classified into category 3. Since these molecules are well expressed in cancer cells, we could consider that they are TAAs. However, they are also expressed in some normal cells.

In the case of mAbs that bound to the Ag classified into category 1, we have to find differences in anti tumor activity between already established therapeutic Abs and our Abs. Even the Abs established against some cancer are effective only to a part of the cancers. Since we isolated nine kinds of anti EGFR mAbs, we examined their *in vivo* anti tumor activity. ERBITUX showed strong anti tumor activity against A431 as well as ACHN in nude mice but did not show anti tumor activity against CCF-RC1 or HT-29. On the other hand, our mAb 048-006 showed strong anti tumor activity against all of the four kinds. Since we have already showed that 048-006 and Erbitux bound to a functionally similar epitope (9), we wonder the orientation of complex formation could be an important factor for anti tumor activity.

In the case of mAbs that bound to the Ag classified into category 2, it seems to be clear for us what we should do. After we examine the ordinary assay for anti tumor activity *in vitro* and *in vivo*, we are going to judge whether clinical examination could be started or not.

Although ordinary researchers are not interested in the Ags classified into category 3 as therapeutic targets, we think these TAAs could be also attractive targets for therapeutic Abs. We have various kinds of mAbs against respective Ags. We comprehensively performed immunostainings against many fresh cancer tissues using multiple mAbs that bound to the same Ag. We found several examples showing tumor-specific staining patterns by some specific clone while both tumor cells and normal cell were equally stained by the other clones. This suggested presence of the tumor-specific epitope different from the epitopes present on both normal and tumor cells that could be distinguished by mAbs. Thus, we believe that some of the Ags classified into category 3 could also become proper targets for therapeutic mAbs.

## Figures and Tables

**Table I:**
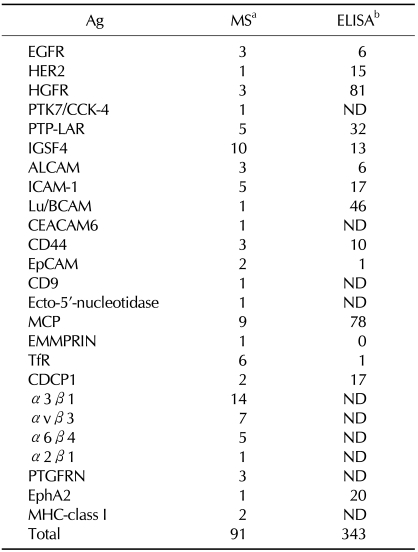
Summary of TAAs identified to date

^a^Number indicates that of different clones identified by MS analysis.^b^Clones identified by MS analysis are not included.

**Table II:**
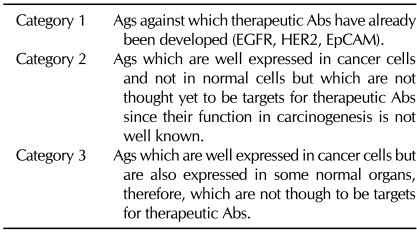
Classification of TAAs for development of therapeutic Abs
